# Assessment of Metal Accumulation by *Arthrospira platensis* and Its Adaptation to Iterative Action of Nickel Mono- and Polymetallic Synthetic Effluents

**DOI:** 10.3390/microorganisms10051041

**Published:** 2022-05-17

**Authors:** Liliana Cepoi, Inga Zinicovscaia, Ludmila Rudi, Tatiana Chiriac, Svetlana Djur, Nikita Yushin, Dmitrii Grozdov

**Affiliations:** 1 Institute of Microbiology and Biotechnology, 1, Academiei Str., MD-2028 Chisinau, Moldova; lilianacepoi@yahoo.com (L.C.); rudiludmila@gmail.com (L.R.); chiriac_tv@yahoo.com (T.C.); djurlana@hotmail.com (S.D.); 2 Joint Institute for Nuclear Research, 6 Joliot-Curie Str., 1419890 Dubna, Russia; ynik_62@mail.ru (N.Y.); dsgrozdov@rambler.ru (D.G.); 3 Horia Hulubei National Institute for R&D in Physics and Nuclear Engineering, 30 Reactorului Str., Magurele, MG-6, 077125 Bucharest, Romania; 4 Institute of Chemistry, 3, Academiei Str., MD-2028 Chisinau, Moldova

**Keywords:** *Arthrospira platensis*, biochemical analysis, proteins, nickel, neutron activation analysis

## Abstract

Cyanobacteria-mediated wastewater remediation is an economical, efficient, and eco-friendly technology. The present work deals with the bioaccumulation performance of *Arthrospira platensis* (Spirulina) grown for four cycles in a medium containing nickel mono- and polymetallic synthetic effluents. The metal uptake by spirulina biomass was evaluated using neutron activation analysis. The effects of effluents on biomass production, protein, and phycobiliprotein content were assessed. Metal accumulation in the biomass depended on the effluent composition and metal ion concentrations. Nickel accumulation in the biomass was directly proportional to its concentration in effluents, and maximum uptake (1310 mg/kg) was attained in the Ni/Cr/Fe system. In the same system, biomass accumulated 110 times more chromium and 4.7 times more iron than control. The highest accumulation of copper (2870 mg/kg) was achieved in the Ni/Cu/Zn/Mo system and zinc (1860 mg/kg)—in the Ni/Cu/Zn/Sr system. In biomass grown in the media loaded with nickel and also chromium, iron, copper, strontium, zinc, and molybdenum, a decrease in productivity (on average by 10%) during the first cycle of cultivation and moderate reduction of protein content (by 15–27%) was observed. The presence of metals in the cultivation media inhibited phycobiliprotein synthesis, especially of phycocyanin, and promoted the synthesis of allophycocyanin. The maximum reduction of phycocyanin content was 77%, and the increase of allophycocyanin content—by 45%. *Arthrospira platensis* may be deemed as bioremediation of nickel-polluted wastewaters of complex composition.

## 1. Introduction

The ever-increasing demands related to the constantly growing population have resulted in an imbalance in the environment caused primarily by pollution with heavy metals and xenobiotics [1]. The danger of environmental contamination with heavy metals is primarily determined by their toxicity even at low concentrations, nonbiodegradability, and accumulation in living organisms [2,3].

Nickel is one of the most concerning metals, widely applied in many industrial processes such as electroplating, forging, ceramics coloring, mineral processing, and the production of stainless steel, batteries, non-ferrous metal coins, metallic alloys, and paints [3,4,5]. Extensive utilization of nickel compounds results in the release of a large volume of industrial effluents and spent nickel-containing products in the aquatic environment, which inevitably leads to an increase in its concentration in various environmental compartments [3]. Globally, the release of nickel in the environment has been estimated to vary from 150,000 to 180,000 metric tons per year [4]. High concentrations of nickel can cause different chronic and acute disorders in humans, related to gene toxicity, neurotoxicity, hepatotoxicity, damage to the kidneys and lungs, gastrointestinal disorders, and cancer, as well as symptoms such as shortness of breath, chest pain, skin dermatitis, and vomiting [3]. The World Health Organization has recommended that nickel concentration in water should be no more than 0.005 ppm [2]. The US EPA has set specific nickel limits for wastewater effluents, which are 2 mg/L for short-term effluent reuse and 0.2 mg/L for long-term effluent reuse [6].

Physical and chemical methods, including chemical precipitation, ion exchange, filtration, coagulation, flocculation, and adsorption, are commonly applied to reduce the levels of heavy metals in wastewater [5,7,8]. These techniques are ineffective or extremely expensive, especially when wastewaters contain a relatively low concentration of metals (1–100 mg/L) dissolved in a large volume of water [8]. The utilization of biological objects is currently receiving wide attention because of their abundant availability, high removal capacity, eco-friendly nature, and low operating costs [5,9,10]. Microorganisms are capable of regulating metal ions and reducing their toxicity through various mechanisms [11].

Cyanobacteria are an extremely diverse group of prokaryotes that perform oxygenic photosynthesis. Due to their adaptive capacity, along with the ability to tolerate extreme conditions, they can be found in many aquatic and terrestrial ecosystems, including environments contaminated with heavy metals [12,13]. Phytoremediation studies performed using microalga and cyanobacteria showed their ability to remarkably reduce the level of toxic chemicals in industrial effluents [13,14,15,16,17,18].

*Arthrospira platensis,* known as Spirulina, is a cyanobacteria industrially grown in different countries and widely used in various fields, including bioremediation processes. The application of *Arthrospira platensis* for bioremediation is primarily determined by the minimal environmental requirements, as this species only needs CO_2_ to intake and the presence of light [19]. Spirulina possesses mechanisms ensuring survival under conditions that differ from the optimal ones. As a model object, Spirulina is used to study the accumulation of various pollutants, including heavy metals. The results are used to develop modern biotechnologies for purifying polluted waters. Moreover, Spirulina not only survives in media with a high content of heavy metals or organic pollutants but also produces a large amount of biomass under these conditions [19].

Spirulina has been shown to accumulate considerable amounts of heavy metals from polymetallic systems of varying compositions and to withstand repeated episodes of pollution [17,18]. In previous studies, it has been shown that *Arthrospira platensis* was able to grow for three cycles in copper-containing and chromium-containing effluents. However, it should be mentioned that in the case of chromium-containing effluents, the biomass survived only at low chromium concentrations [17,18]. Nickel sorption and bioaccumulation by living organisms have not received as much attention in the literature as the removal of other metals, probably because of the relatively low uptake capacities achieved [20].

The present study aimed to trace the effect of nickel mono- and polymetallic synthetic effluents on the cyanobacteria *Arthrospira platensis* during iterative action to investigate its applicability as a renewable bioremediator.

## 2. Materials and Methods

### 2.1. Chemicals

The salts (analytical grade) used to prepare synthetic effluents were Ni(NO_3_)_2_·6H_2_O, Cu(NO_3_)_2_·2.5H_2_O, Zn(NO_3_)_2_·6H_2_O, Sr(NO_3_)_2_, Na_2_MoO_4_·2H_2_O, CrO_3._ All chemicals used in the present study were obtained from Sigma Aldrich, Germany.

### 2.2. Synthetic Effluents

Wastewaters discharged into freshwater bodies are usually characterized by the presence of more than one toxic or potentially toxic substance. Therefore, compared to single metal studies, examining the effects of multi-metal solutions better represents actual environmental conditions [21]. Based on data for real wastewater presented previously [22,23], for the present work, four synthetic effluents in three concentrations variants were prepared, and their chemical composition and metal ion concentrations are given in Table 1.

### 2.3. Experiment Design

The *Arthrospira platensis (A. platensis)* strain CNMN-CB-02 was obtained from collecting non-pathogenic microorganisms at the Institute of Microbiology and Biotechnology (Chisinau, Moldova). For obtaining inoculum for the first cultivation cycle the cyanobacterium was grown in a mineral medium with the following composition: macroelements (g/L): NaNO_3_—2.5; NaHCO_3_—8.0; NaCl—1.0; K_2_SO_4_—1.0; MgSO_4_·7H_2_O—0.2; CaCl_2_—0.024; FeSO_4_·7H_2_O—0.01; microelements (mg/L): H_3_BO_3_—2.86; MnCl_2_·4H_2_O –1.81; ZnSO_4_·7H_2_O—0.22; CuSO_4_·5H_2_O—0.08; MoO_3_—0.015. The same medium was used for the experiment but without the microelements included in the systems.

The experiment was carried out in Erlenmeyer flasks with a working volume of 700 mL. Spirulina was cultivated at pH of the medium 8–10, temperature 28–30 °C, light intensity of 55 μmol photons m^−2^ s^−1^, continuous illumination, slow periodic 30-min shaking 2 times per 24 h with an interval of 12 h with a frequency of 100 r.p.m. (Heidolph UNIMAX 1010, Heidolph, Germany). The amount of inoculum was 0.4 g/L.

On the fifth day, metal solutions were added to the medium according to Table 1. On the sixth day, the experiment (I cycle of cultivation) was stopped. Biomass suspension was divided into three portions: 100 mL for biochemical tests, 300 mL for neutron activation analysis, and 300 mL for a new inoculum. Each portion of the biomass was centrifuged. The biomass portion for analysis was washed with distilled water. The biomass for the biochemical tests was standardized with distilled water to a concentration of 10 mg/mL. The biomass for neutron activation analysis was dried at 100 ± 2 °C.

The procedure was repeated for the second, third, and fourth (II-IV) cultivation cycles. The control biomass was cultivated under the same conditions without adding metal ions to the cultivation medium.

### 2.4. Neutron Activation Analysis (NAA)

The accumulation of metal ions by *A. platensis* biomass was assessed using neutron activation analysis (NAA) at the REGATA facility of the IBR-2 reactor (JINR, Dubna, Russia). To determine the copper content, samples were irradiated for 3 min at a thermal neutron flux of 1.2 × 10^12^ n cm^−2^ s^−1^ and measured for 15 min. The content of nickel, zinc, chromium, iron, strontium, and molybdenum was determined after sample irradiation for 3 days at a neutron flux of 1.1 × 10^11^ n cm^−2^ s^−1^. Then samples were repacked and measured after 4 and 20 days for 30 min and 1.5 h, respectively. NAA data processing and calculating element concentrations were performed using Genie2000 and the software “Concentration” developed in JINR.

The quality control of the measurements was assured by simultaneous irradiation of the samples and standard reference materials. The difference between the experimentally obtained and certified values did not exceed 10%.

### 2.5. Biochemical Analysis

Biomass quantity was determined spectrophotometrically (Spectrophotometer T80 UV/VIS, PG Instruments Ltd., Alma Park, Woodway Lan, Wibtoft Leicestershire, UK) by measuring the absorbance of the spirulina suspension at 750 nm before centrifugation.

The protein content was measured according to the Lowry method based on the Biuret reaction and the reaction with the Folin-Ciocalteu reagent. The phycobiliprotein content was determined spectrophotometrically as per the method described by Siegelman and Kycia. The results were expressed in % biomass. A detailed description of the biochemical test is given in our previous study [24].

### 2.6. Statistical Analysis

All experiments and measurements were performed in triplicate. One-way analysis of variance (ANOVA) was performed using Statistica 10 (Student’s *t*-tests). Data in all Tables and Figures are presented as mean value ± SD.

## 3. Results and Discussion

Compared to other prokaryotes, the photosynthetic machinery in cyanobacteria imposes higher demand for metals. Thus, Fe is necessary for all three photosynthetic electron transfer chain complexes, Mn for the water-splitting complex, Cu for plastocyanin and cytochrome c oxidase, Mg for chlorophylls, and Zn for carbonic anhydrase [12]. Nickel, along with Zn, Cu, Mo, and Fe, are essential nutrients for microorganisms since they participate in various cellular processes [25]. According to literature data, nickel is present in the main part of commercially available spirulina products. Thus, in 23 samples of spirulina produced in Australia, the USA, Great Britain, Japan, India, Canada, and New Zealand, its content varied from 0.21 to 4.6 mg/kg d.w. [8]. In the present study, nickel content in control spirulina biomass was 11.8 mg/kg.

### 3.1. Ni System

**Figure 1:**
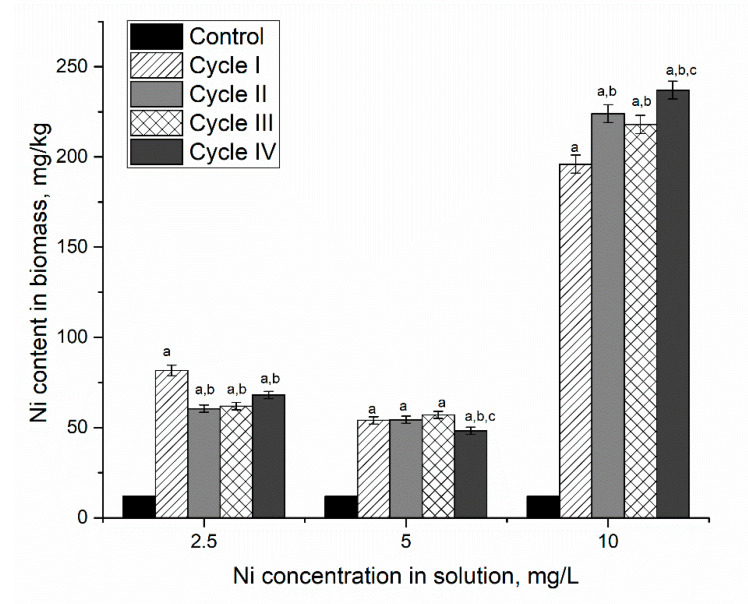
Accumulation of nickel ions in spirulina biomass during repeated cultivation in a medium containing nickel ions at concentrations of 2.5–10 mg/L. a: *p* < 0.001 for the difference between experimental and control samples; b: *p* < 0.001 for the difference with the first cycle; c: *p* < 0.001 for the difference with the second cycle.

The addition of nickel ions at different concentrations (according to Table 1) to the cultivation medium increased their content in the biomass (Figure 1). That is in agreement with Rugnini et al. [21]. However, the efficiency of metal uptake depended on the cycle of cultivation. Thus, in the system containing 2.5 mg/L of nickel ions, the highest accumulation of nickel ions was noticed during the first cultivation cycle, when the biomass accumulated 7 times more nickel ions than control. Over the next three cultivation cycles, their content in biomass was 5.1–5.7 times higher than control but lower than in the first cycle. At nickel concentration in solution 5 mg/L, its content in biomass was 4.6–4.7 times higher than in control. Thus, the accumulation of nickel ions by *Spirulina platensis* biomass at concentrations in the solution of 2.5 and 5 mg/L was almost at the same level. Increased nickel concentration up to 10 mg/L in solution led to a significant increase (*p* < 0.001) of its content in biomass, 17–20 times in comparison with control. The most pronounced increase was observed in the first cycle of cultivation, by 1560%, with respect to control. In the next three cultivation cycles, the increase in nickel uptake was less significant. Thus, in the second cycle, it was 14% higher than in the first cycle. In the third cycle, a slight decrease (by 2.3%) was noticed, while in the fourth cycle, an increase of 8.7% took place.

Cyanobacteria *Anabaena cylindrica*, *Anabaena flos-aquae*, and *Nostoc* sp. accumulated 2–6 mg/g of nickel at a 10 mg/L concentration in solution [26]. The uptake of nickel ions in the present study was lower compared to values presented by Rugnini et al. [21] for two cyanobacteria, *Chlorella vulgaris* and *Desmodesmus* sp., although the range of the studied concentrations in both studies was very similar. The maximum accumulation of nickel by *Synechococcus* sp. IU 625 occurred on the fifth day of biomass growth; however, nickel was undetectable inside the cells on day 11. The release of nickel ions from biomass can be explained by overexpression of the smtA gene and other alternative mechanisms. At the same time, it should be mentioned that *Synechococcus* sp. IU 625 demonstrated high resistance to nickel ions, maintaining high biomass growth rates at nickel concentrations in the cultivation medium of 10 and 25 mg/L [27].

**Figure 2:**
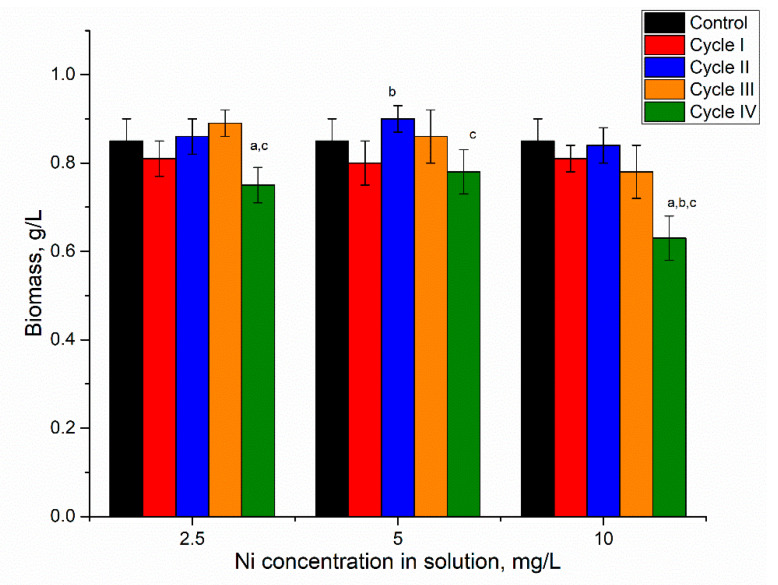
The amount of biomass in spirulina cultivated in a medium containing nickel ions at concentrations of 2.5–10 mg/L during four cycles. a: *p* < 0.001 for the difference between experimental and control samples; b: *p* < 0.001 for the difference from the first cycle; c: *p* < 0.001 for the difference from the second cycle.

The addition of nickel ions to the medium in concentrations of 2.5–10 mg/L for the first cultivation cycle did not significantly affect spirulina biomass accumulation (Figure 2).

At the end of the first cultivation cycle, the biomass was reduced insignificantly by 5–6%, and this response was the same for all applied nickel concentrations. In the next two cycles, in all analyzed systems, the amount of biomass returned to control values. At the end of the fourth cycle, the amount of biomass was reduced in all analyzed systems, as the resulting values were 0.73, 0.78, and 0.75 g/L for nickel concentrations at 2.5, 5, and 10 mg/L in the medium, respectively. For a nickel concentration of 2.5 mg/L, biomass reduction in the fourth cultivation cycle was significant (*p* < 0.001) compared to the second and third cycles. At a nickel concentration of 10 mg/L, the decrease in the fourth cycle was significant (*p* < 0.001) compared to control and other cycles of biomass growth, while at a concentration of 5 mg/L the reduction was insignificant. Thus, the reported in [28] dose-dependent decrease of *Spirulina indica*, *Spirulina maxima*, and *Spirulina platensis* growth with the increase of nickel concentration in the medium was not observed in the present work even at repeated cultivation.

*Spirulina platensis* is one of the richest protein sources of microbial origin containing 46–63% dry biomass (DB) of this nutrient [29]. Proteins serve multiple purposes as structural components, enzymes, membrane transporters, signaling molecules, or regulatory factors. In cyanobacteria, some proteins as, for example Psb27, are important in photosystem II (PSII) repair, while others, like PetP, are involved in the stress adaptation of the photosynthetic electron transport [30].

**Figure 3:**
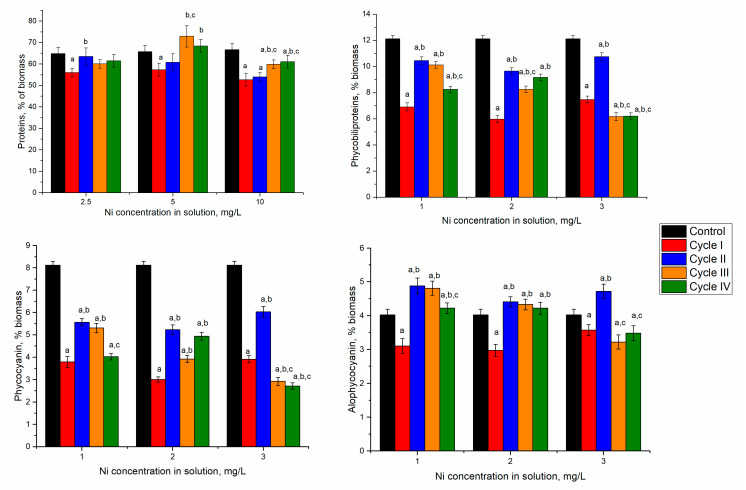
The content of proteins and phycobiliproteins in spirulina biomass cultivated in a medium containing nickel ions at 2.5–10 mg/L over four cycles. a: *p* < 0.001 for the difference between experimental and control samples; b: *p* < 0.001 for the difference from the first cycle; c: *p* < 0.001 for the difference from the second cycle.

Spirulina’s response to nickel ion effects in the first cultivation cycle was manifested by reducing biomass and protein content. However, in the next cycles, spirulina adaptation to the new conditions of growth was observed (Figure 3).

In spirulina biomass growing in the media containing 2.5 and 5 mg/L of nickel ions, at the end of the first cultivation cycle, the content of proteins in biomass significantly decreased by 12–15% (*p* < 0.001). Nickel concentration of 10 mg/L led to the reduction of protein content by 19%. This could be due to the stress caused by the nickel ions present in the culture medium. In microorganisms, nickel toxicity is expressed in the inhibition of metalloproteins, interaction with enzyme active site His or Cys residues, and enhancement of oxidative stress [31]. Starting with the second cultivation cycle, at all nickel concentrations, the protein content of biomass increased in comparison with the first cycle. At a nickel concentration of 2.5 mg/L in the medium, the content of proteins over the next three cycles almost reached control values. The difference between the second–fourth cycles and the first cycle was statistically significant (*p* < 0.001). At a nickel concentration of 5 mg/L in the medium, the maximum amount of proteins was attained in the third cultivation cycle. This value was significantly higher (*p* < 0.001) than the first two cycles, but it did not differ essentially from control.

The increase in protein content can be explained by the excretion of nickel ions from the cell. Huertas et al. [27] showed that an excess of nickel that could cause a perturbation of protein function is counterbalanced by the presence of efflux pumps that expel it out of the cell. The slight increase or decrease of nickel content compared to the first cycle confirmed this fact.

In cyanobacteria, phycobiliproteins, large water-soluble supramolecular protein aggregates serve as major accessory pigments in photosynthesis. They can be divided broadly into three classes: phycoerythrin, phycocyanin, and allophycocyanin, based on their spectral properties [32]. Spirulina contains two of these pigments—phycocyanin and allophycocyanin. In all experimental variants, the amount of phycobiliproteins was significantly lower (*p* < 0.001) compared to control (Figure 3).

In the experimental variants with nickel concentrations of 2.5 and 5 mg/L, at the end of the first cultivation cycle the content of total phycobiliproteins in biomass was reduced by 43–51%. Consecutive cultivation in the second cycle was beneficial for the culture. The amount of phycobilin increased by 51% and 61% compared to the first cycle. However, it was lower by 13 and 20% compared to control biomass. The tendency to restore phycobiliprotein content was maintained for two further cultivation cycles. At the end of the fourth cycle, the phycobiliprotein content increased by 19 and 53%, respectively, compared to values obtained in the first cycle. At the same time, it was lesser than control by 32% and 24%, respectively.

In the case of the application of 10 mg/L, the decrease of the phycobiliprotein content by 38% in the first cycle was followed by an increase of 44% in the second cycle, and in the next two cycles it remained slightly lower compared to the first cycle. Thus, the introduction of nickel ions in the culture medium induced the inhibition processes of phycobiliprotein synthesis, regardless of the applied concentration. A dose-dependent decrease of protein content with an increase in nickel concentration was observed for Spirulina strains [33].

Significant reduction in phycobiliprotein content was mainly determined by decreasing phycocyanin by more than 50% in the first and fourth cycles of biomass growth (Figure 3). By adding 2.5 mg/L of nickel in the first cycle, the phycocyanin content decreased by 53%, compared to 23% in control and allophycocyanin. A recovery attempt was observed in the second and third cycles, but phycocyanin values remained low (30–35% below the control values). In the case of allophycocyanin, its content in the second cycle increased significantly by 21% (*p* < 0.001) compared to control and was maintained in the third cycle. In the last cycle, the allophycocyanin content was at the control level.

At the introduction of 5 mg/L of nickel in the cultivation medium and the end of the first cultivation cycle, phycocyanin content decreased by 63% and allophycocyanin by 26% compared to control. The tendency to rectorate the phycocyanin content during repeated cultivation in the medium containing nickel ions failed. Its content in the fourth cycle was 39% lower than the control value. At the same time, the content of allophycocyanin returned to the control values. The least favorable situation was observed at a nickel concentration of 10 mg/L when in the first cycle of cultivation, the phycocyanin content was reduced by 52% and at the end of the fourth cycle by 66%. The content of allophycocyanin was significantly lower (*p* < 0.001) compared to control in the first, third, and fourth cycles and higher in the second cycle. Thus, upon repeated contact with the metal in the second cycle, the spirulina culture restored its allophycocyanin level, but the repeated action of the pollutant eventually leads to a stable reduction of the pigment content.

It is known that both phycocyanin and allophycocyanin bind heavy metal ions differentially, whereas phycocyanin has a higher affinity for them. Thus, metal binding preferentially to phycocyanin can be one of the reasons explaining the significant decrease in their content [34]. The same authors reported that silver and copper ions show minimal binding to allophycocyanin, as they induce structural changes such as a decrease in absorbance and fluorescence of phycocyanin.

### 3.2. Ni/Cr/Fe System

**Figure 4:**
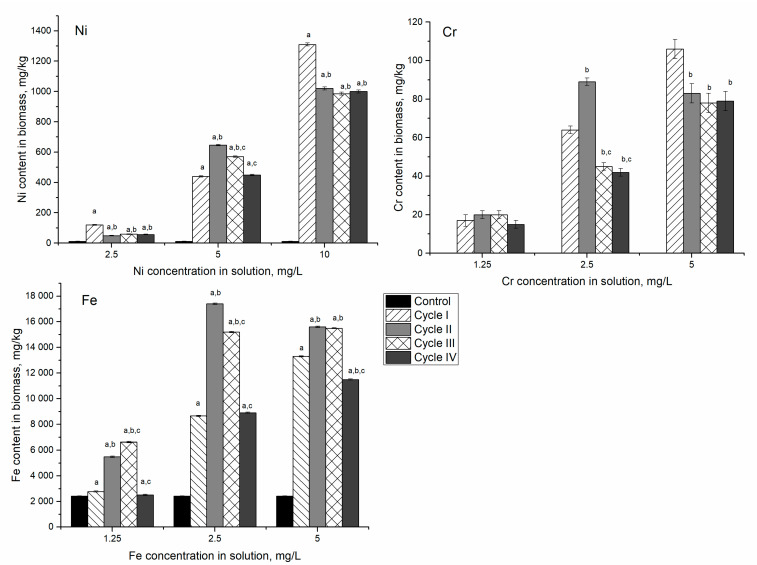
Accumulation of metal ions in spirulina biomass during its repeated cultivation in the medium containing metal ions at concentrations: nickel 2.5–10 mg/L, chromium and iron 1.25–5 mg/L. a: *p* < 0.001 for the difference between experimental and control samples; b: *p* < 0.001 for the difference from the first cycle; c: *p* < 0.001 for the difference from the second cycle.

In the Ni/Cr/Fe system at nickel concentration in the solution of 2.5 mg/L, after the first cycle of cultivation, nickel content in biomass was 10 times higher than control (Figure 4). In the next three cycles, its content in biomass decreased approximately twice and was almost on the same level. At nickel concentration in solution 5 mg/L, its content in biomass increased 37–55 times.

The most significant increase was noticed in the second cycle when nickel content in biomass exceeded control value by 83–111 times. At nickel concentration of 10 mg/L, the most pronounced increase was observed in the first cultivation cycle, then in the next three cycles, it decreased by 22–25% compared to the first cycle. The amount of Ni accumulated by the biomass in Ni/Cr/Fe systems was 1.5–11 times higher than in the system containing only nickel ions.

Chromium content in control biomass was below the detection limit of NAA. However, its content in the system containing Cr(VI) ions varied from 17 to 110 mg/kg. At a chromium concentration of 1.25 mg/L, the content of the element accumulated in biomass was approximately the same during all cultivation cycles. At a concentration of 2.5 mg/L in the first cycle of cultivation, the spirulina biomass accumulated 3 times more chromium compared to the medium with a concentration of 1.25 mg/L. In the second cycle, this amount increased to 90 mg/kg. In the next two cycles, the amount of chromium accumulated decreased twice compared to the second cycle. At chromium concentration of 5.0 mg/L, the highest amount of the element was accumulated in the first cycle of cultivation, after which it decreased and remained at the same level for the next three cycles.

Iron accumulation in biomass also increased with the increase of its concentration in the solution. With the addition of 1.25 mg/L of iron ions, the content in biomass continuously increased over three cultivation cycles, and then it decreased to the level of control. At higher Fe concentrations, 2.5 and 5 mg/L, the same pattern was observed. However, in these experimental variants, after four cultivation cycles, Fe content in biomass was 3.7 and 4.7 times higher than in control, respectively. As in the case of the Ni system, the removal of metal ions from cells to decrease the toxic effect on spirulina was observed.

**Figure 5:**
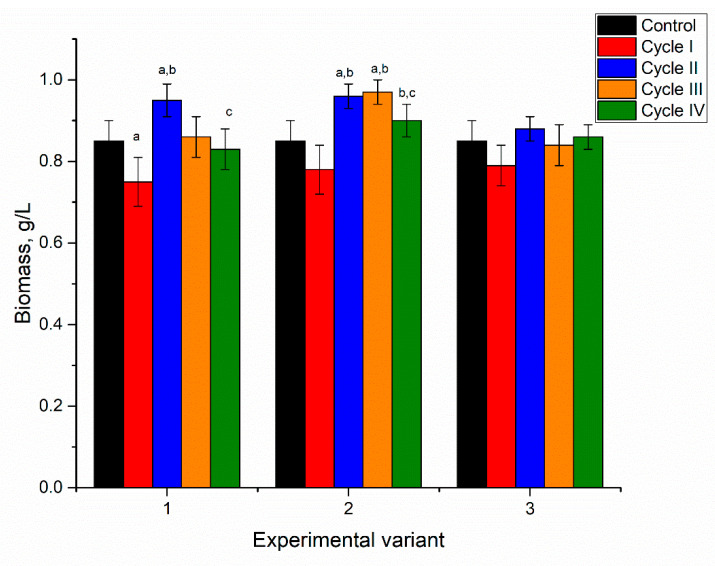
The amount of spirulina biomass during repeated cultivation in a medium containing metal ions at concentrations: nickel 2.5–10 mg/L, chromium and iron 1.25–5 mg/L. a: *p* < 0.001 for the difference between experimental and control samples; b: *p* < 0.001 for the difference with the first cycle; c: *p* < 0.001 for the difference with the second cycle.

For spirulina grown in the medium containing ions of three metals, nickel, chromium, and iron, at the end of the first cycles in all experimental variants, the biomass decreased by 7–12%. However, only in the case of the first experimental variant was this difference significant (*p* < 0.001) compared to control. In the other cases, there was a tendency for reduced biomass productivity (Figure 5). In the next three cycles, biomass was on the level of control or overpassed it. Thus, in the first experimental variant, the amount of biomass in the second cycle was significantly higher than control. In the second experimental variant, the amount of biomass was higher than control in the second and third cycles. In the rest of the cases, the obtained values were at the level of control.

While assessing the effect of nickel concentration on biomass, it was noticed that at concentrations of 2.5 mg/L and 10 mg/L, the maximum metal accumulation took place in the first cycle of cultivation, leading to decreased spirulina productivity. In the next two cycles, nickel content in biomass was reduced by 2 and 1.3 times, respectively, while biomass increased by 12–14% compared to control. The highest accumulation of iron, in all experimental variants, was observed in the second and third cycles. Thus, dependence between the accumulation of Fe and biomass production can be assumed, although it occurred at levels characteristic of the strain under study. At the same time, it should be noted that in the nickel mono-system, in the first and third experimental variants, the amount of biomass was significantly lower than control, while in the analyzed poly-systems, this effect was not observed.

**Figure 6:**
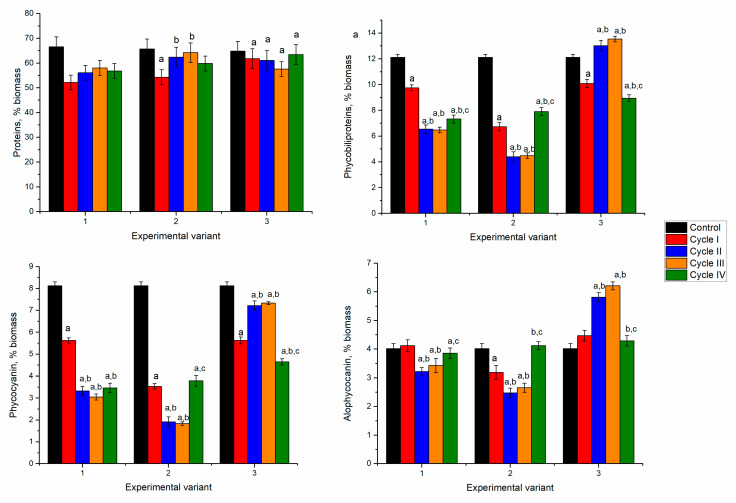
The content of proteins and phycobiliproteins in spirulina during repeated cultivation in the medium containing metal ions at concentrations: nickel 2.5–10 mg/L, chromium and iron 1.25–5 mg/L. a: *p* < 0.001 for the difference between experimental and control samples; b: *p* < 0.001 for the difference from the first cycle, c: *p* < 0.001 for the difference from the second cycle.

Protein content in the first experimental variant (at a Ni concentration of 2.5 mg/L) was not significantly affected by metal ions in the cultivation medium during repeated cultivation (Figure 6).

For the second and third variants (5 mg/L and 10 mg/L), the content of proteins at the end of the first cycle was significantly reduced by 17–20% (*p* < 0.001). A tendency to restore the protein content towards the end of the fourth cycle was observed. In the second experimental variant, the amount of protein in spirulina biomass at the end of the second–fourth cycles did not differ significantly from control. The situation was different in the third experimental variant. Although there was a tendency to restore the protein content in cycles II-IV, it remained significantly lower than control.

In the first experimental variant, the content of phycobiliproteins decreased insignificantly, by 19% in the first cycle of cultivation and by 39–46% in the next three cycles (Figure 6). The phycocyanin content decreased drastically, by 31–62%, compared to control. The allophycocyanin content did not change significantly. The maximum decrease of 19% took place in the second cycle.

In the second experimental variant, a critical decrease in the content of phycobiliproteins took place in the second and third cycles. Their value was 63% lower than the control. At the end of the fourth cultivation cycle, the content of phycobiliproteins remained low, the value 35% lower than control. The phycocyanin content decreased by 56% in the first cycle and 76–77% in the second and third cycles. The allophycocyanin content was reduced by 20–38% during the first three cultivation cycles and returned to normal values in the fourth cycle.

It is interesting that in the third experimental variant, the content of phycobiliproteins changed less during repeated cultivation and decreased by 26% compared to control in the fourth cycle. Content of phycocyanin in the first and fourth cycles was also significantly reduced, by 31–43% (*p* < 0.001), while the allophycocyanin content remained almost unchanged. In the second and third cycles, the content of phycobiliproteins and phycocyanin did not change, and an increase in the content of allophycocyanin by 45% was noted. In our previous study, we showed that in spirulina biomass grown in a Cr/Fe system, the content of phycobiliproteins was reduced by approximately 70% [18]. Since Bellamy-Carter and co-authors [34] reported little to no binding of iron ions by phycobilins, it can be suggested that nickel and chromium are responsible for the decrease of phycobiliproteins.

### 3.3. Ni/Cu/Sr/Zn System

**Figure 7:**
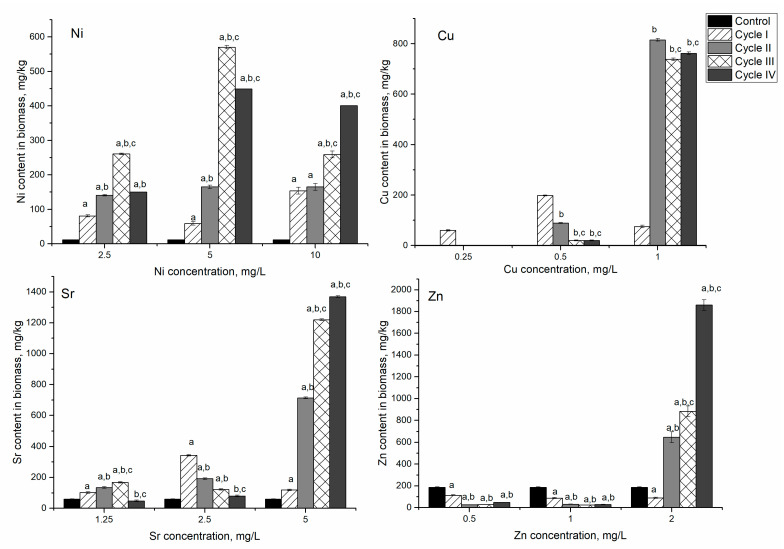
Accumulation of metal ions in spirulina biomass during repeated cultivation in the medium containing metal ions at concentrations: nickel 2.5–10 mg/L, copper 0.5–1 mg/L, strontium—1–5 mg/L, and Zn—0.5–2 mg/L. a: *p* < 0.001 for the difference between experimental and control samples; b: *p* < 0.001 for the difference from the first cycle; c: *p* < 0.001 for the difference from the second cycle.

In the Ni-Cu-Sr-Zn system in the first experimental variant, the nickel content in biomass increased during three cycles of cultivation by 6.9–22 times compared to control. In the fourth cycle, it declined to the level of the second cycle (Figure 7). The level of nickel accumulation was higher compared to the monometallic system, where the maximum amount of nickel, 80 mg/kg, was accumulated in the first cycle; and compared to the Ni/Cr/Fe system, where biomass accumulated up to 100 mg/kg of nickel in the first cycle of cultivation. In the analyzed system, maximum accumulation of nickel (250 mg/kg) was achieved in the third cycle, and it was 2.5–3.0 higher than the Ni and Ni/Cr/Fe systems.

Copper was detected in biomass only after the first cycle of cultivation. Accumulation of strontium by biomass was similar to the nickel uptake. It increased over three cultivation cycles, and then, in the fourth cycle, it decreased below the control level. Zinc was the only element whose content in biomass continuously decreased from 186 mg/kg in control biomass to 23 mg/kg after four cycles of cultivation.

In the second experimental variant, maximum metal accumulation was achieved in different cycles: nickel (646 mg/kg) in the third cycle of cultivation, and copper (192 mg/kg) and strontium (343 mg/kg) in the first cycle. Then their content in biomass decreased in comparison to the maximum accumulated amount, but it was higher than in control biomass. For zinc, it was noted the same pattern as in the first experimental variant, as its content in biomass decreased by 86% compared to control. The decrease of zinc content in biomass can be explained through replacement by other metal ions, in particular copper, present in the analyzed system. Copper displays a high affinity for metalloproteins, and if equivalent quantities of all divalent metals were present, proteins would probably all bind copper [35]. The decrease in zinc content can also be explained by the inhibition of zinc metalloenzymes by nickel [36]. The reduction of strontium content can also be explained by its replacement with nickel ions [33]. In general, the content of nickel in biomass increased 5–55 times, of Cu 20–198 times, and of Sr 1.3–5.7 times.

An interesting pattern was observed in the third experimental variant. Nickel, strontium, and zinc content in biomass continuously increased over the cycles. Thus, the amount of nickel accumulated by biomass increased from 1200% in the first cycle to 3300% in the fourth cycle compared to control. Zinc behavior in the third experimental variant was different from the other experiments. It was characterized by the increase of its content in biomass by a factor of 10 with respect to control. The maximum amount of copper was accumulated by the biomass in the second cycle of cultivation (815 mg/kg), and then it slightly decreased in the next two cycles of cultivation (approximately 9%). The possible mechanisms of cyanobacteria resistance to heavy metals include complexation and active efflux [37]. Accumulation of lesser nickel content compared to the second experimental variant indicated that its efflux is the main mechanism of spirulina resistance. Continuous accumulation of strontium and zinc suggested their binding to metallothioneins, which are synthesized under heavy metal stress [37]. In the case of copper, both mechanisms of cyanobacteria resistance are possible.

**Figure 8:**
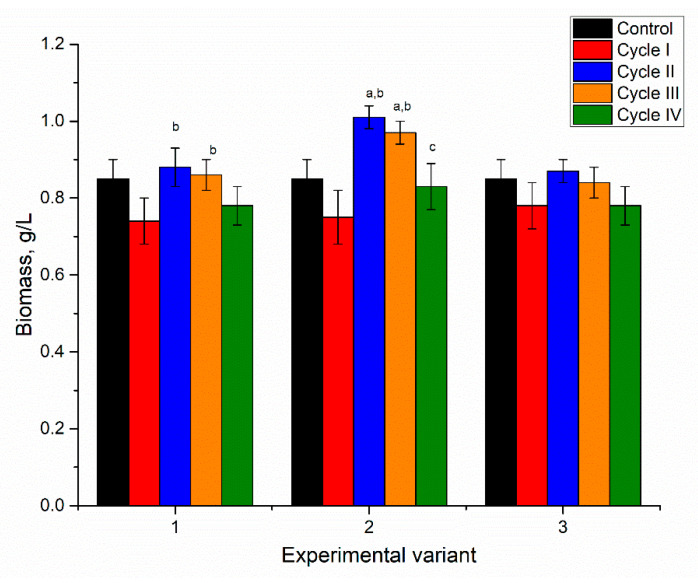
The amount of biomass in spirulina during its repeated cultivation in the medium containing metal ions at concentrations: nickel 2.5–10 mg/L, copper 0.5–1 mg/L, strontium—1–5 mg/L, and zinc—0.5–2 mg/L. a: *p* < 0.001 for the difference between experimental and control samples; b: *p* < 0.001 for the difference from the first cycle; c: *p* < 0.001 for the difference from the second cycle.

Accumulation of nickel, copper, zinc, and strontium in spirulina did not have a negative impact on biomass production during consecutive cultivation in a metal-loaded medium (Figure 8). In all experimental variants, the culture’s response to the introduction of metals in the cultivation medium was similar to the Ni system. It was characterized by a decrease in biomass by 10% compared to control at the end of the first cycle. This difference was statistically insignificant.

In the first and third experimental variants over the next three cycles, the biomass was maintained at the level of control. In the second experimental variant, there was an increase in biomass productivity by 14–18% at the end of the second and third cultivation cycles. The maintenance of high biomass productivity under loading of chemical elements indicated adaptation of spirulina biomass to the new growth conditions.

**Figure 9:**
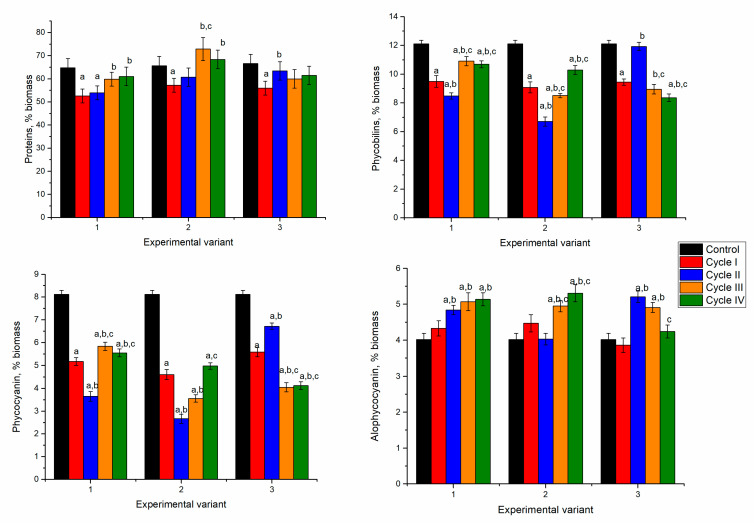
The content of proteins and phycobilins in spirulina biomass during repeated cultivation in the medium containing metal ions at concentrations: nickel 2.5–10 mg/L, copper 0.5–1 mg/L, strontium—1–5 mg/L, and Zn—0.5–2 mg/L. a: *p* < 0.001 for the difference between experimental and control samples; b: *p* < 0.001 for the difference from the first cycle; c: *p* < 0.001 for the difference from the second cycle.

The content of proteins in spirulina grown with the addition of Ni/Cu/Sr/Zn was also insignificantly affected by the presence of metal ions (Figure 9). As in the case of biomass productivity, in all experimental variants, the content of proteins in biomass at the end of the first cycle decreased by 13–15% with respect to control. Restoration of the protein content took place in consecutive cultivation cycles and reached the level of control.

The addition of elements according to the first experimental variant resulted in decreased content of phycobiliproteins at the end of the first cycle by 21%. It maintained its low in the second cycle, reaching values approximately equal to control, but in the third and fourth cycles it was significantly lower. In the second experimental variant, the critical content of phycobiliproteins was obtained during the second cycle of cultivation, when their content was 44% lower than control. At the end of the fourth cycle of cultivation, the phycobiliprotein content was restored, and the values were 15% lower than control. In the case of the third experimental variant, in the first cycle, the phycobilin content decreased by 22% compared to control. In the second cycle, their content was approximately equal to control. However, in the next cycles, it was reduced again to the level of the first cycle.

The significant reduction in phycobiliprotein content was caused by decreased phycocyanin quantity. In the first experimental variant, during the first two cultivation cycles, the phycocyanin content decreased by 63% compared to the control, while in the third and fourth cycles, its content increased and reached a value 32% lower than control. The allophycocyanin content increased over the four cultivation cycles by 20–28%.

The same tendency was observed in the second experimental variant. In the first two cycles, the maximum reduction of phycocyanin was 67%. In the next two cycles, it started to increase, and by the end of the fourth cycle, it was lower than control by 39%. The allophycocyanin content increased during cultivation by 23–32%.

A different response was established for the third experimental variant, in which, in the first cycle of cultivation, the phycocyanin content decreased by 31% below the control. However, it tended to get restored in the second cycle, but it was again reduced in the next two cycles. Allophycocyanin, which content in the first cycles was on the level of control, increased in the next cycles by 30% compared to control. Zinc, copper, and nickel can be responsible for decreasing phycobiliprotein content. Reducing phycobiliprotein content in the presence of zinc, copper, and nickel ions in the cultivation medium were also reported in other studies [17,38,39].

### 3.4. Ni/Cu/Zn/Mo System

**Figure 10:**
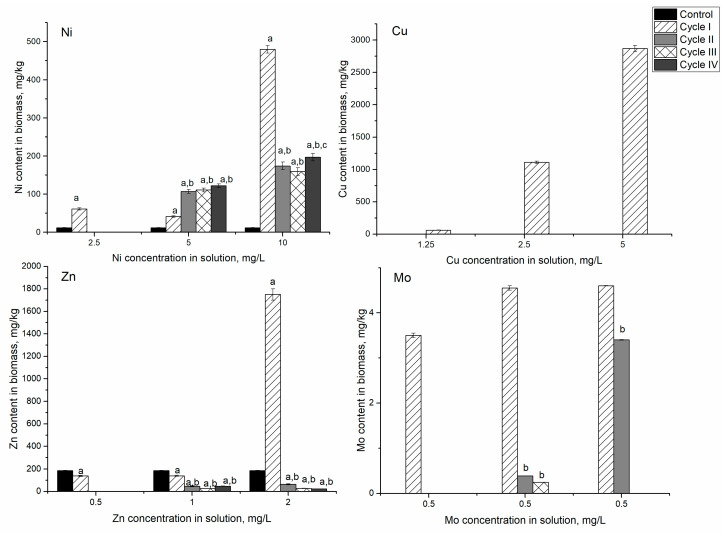
Accumulation of metal ions in spirulina biomass during repeated cultivation in the medium containing metal ions at concentrations: nickel 2.5–10 mg/L, copper 1–5 mg/L, zinc 0.5–2 mg/L, and molybdenum 0.5 mg/L. a: *p* < 0.001 for the difference between experimental and control samples; b: *p* < 0.001 for the difference from the first cycle; c: *p* < 0.001 for the difference from the second cycle.

In the Ni-Cu-Zn-Mo system, in the first experimental variant, spirulina biomass survived only for one cycle (Figure 10). At the end of the first cycle of cultivation, spirulina accumulated 40% of protein, an amount insufficient for biomass growth recovery. One of the main reasons for such an effect can be the low zinc content in the system.

As a result, the nickel content in biomass increased 5 times, the content of copper 59 times, and the quantity of molybdenum was 3.5 mg/kg. The zinc content in biomass decreased by 25% compared to control.

In the second experimental variant, even though the metal concentrations in the solution were higher, the biomass could grow for four cycles. Additionally, the solution of specified chemical composition resulted in increased copper content in biomass by a factor of 1110 in the first cycles of cultivation. In the next cycles, Cu was not detected in biomass. The same pattern was marked for molybdenum, which content was reduced from 4.5 mg/kg (first cycle of cultivation) to an undetectable level (fourth cycle). The zinc content in biomass continuously decreased over the cycles compared to control, while nickel increased (3–5/5.1 times). Adaptation to high metal concentration in cyanobacteria is manifested through several mechanisms: the ability to excrete to the media-heavy metal ligands, like siderophores or extracellular polymeric substances, the production of metallothioneins, or the induction of metal transporters [28].

In the third experimental variant, maximum accumulation of all metal ions present in the mixture took place in the first cultivation cycle: 40 times for nickel, 2870 times for copper, 9.4 times for zinc, and 4.6 mg/kg for molybdenum. In the next three cycles, a drastic decrease in metal uptake was noticed. By the fourth cycle, copper and molybdenum were not detected in biomass, zinc content was lower by 88% than in control, while nickel was 17 times. Nickel adversity in microorganisms is mostly due to its ability to interfere with or replace other essential minerals, such as iron, zinc, copper, calcium, magnesium, molybdenum, iodine, potassium, and sodium in cells [33]. The observed reduction of the levels of zinc, copper, and molybdenum in biomass support this fact.

**Figure 11:**
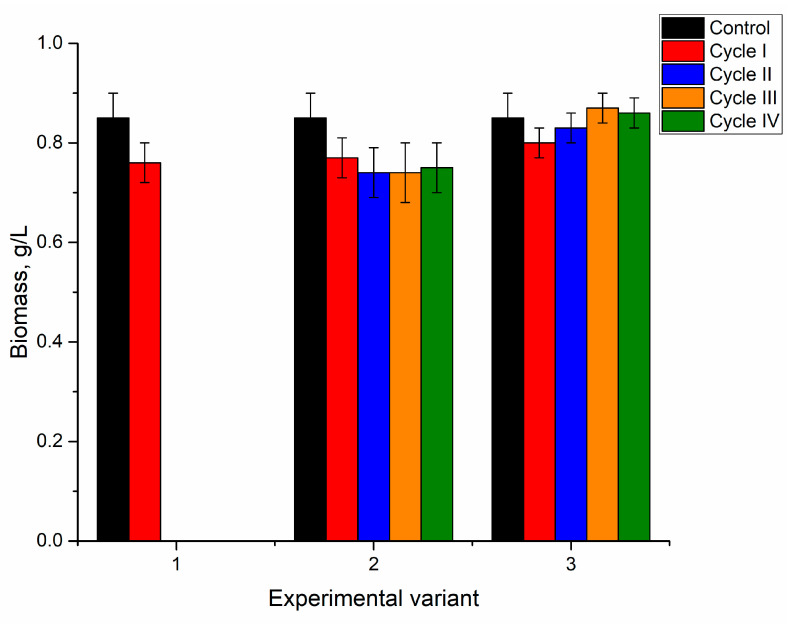
The amount of biomass in spirulina during repeated cultivation in the medium containing metal ions at concentrations: nickel 2.5–10 mg/L, copper 1–5 mg/L, zinc 0.5–2 mg/L, and molybdenum 0.5 mg/L.

As mentioned previously, in the first experimental variant, the spirulina biomass was able to grow only for one cycle, and the biomass was 12% lower (statistically insignificant) than the control (Figure 11). In this case, a correlation between productivity and the accumulation of metals in biomass cannot be determined. In the second experimental variant, the biomass productivity was lower by 10–13% (statistically insignificant) than in control. The biomass content obtained in the third experimental variant did not appreciably change over the repeated cultivation in a metal loaded medium.

**Figure 12:**
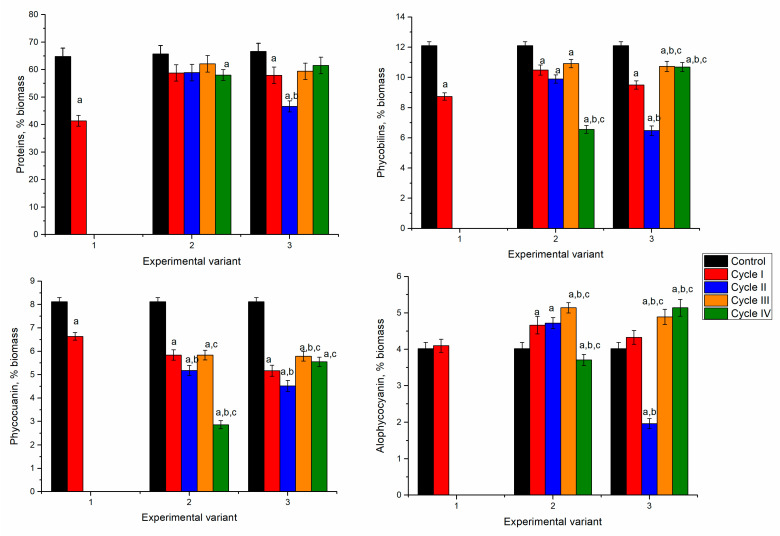
The content of proteins and phycobiliproteins in spirulina during repeated cultivation in the medium containing metal ions at concentrations: nickel 2.5–10 mg/L, copper 1–5 mg/L, zinc 0.5–2 mg/L, and molybdenum 0.5 mg/L. a: *p* < 0.001 for the difference between experimental and control samples; b: *p* < 0.001 for the difference from the first cycle; c: *p* < 0.001 for the difference from the second cycle.

The biomass obtained in the first experimental variant at the end of the first cycle of cultivation contained 41.4% of proteins, which was 37% less compared to the control sample (Figure 12). In the second experimental variant, the values of the protein content in biomass over the four cycles of repeated cultivation were relatively stable and slightly below the control level, and the differences were statistically insignificant. In the third experimental variant, the metal ions negatively influenced protein synthesis during two consecutive cultivation cycles. The value initially decreased by 27% but then returned to control during the third and fourth cultivation cycles.

The content of phycobiliproteins in the analyzed system changed differently. Thus, in the first experimental variant, it decreased by 12%. In the second experimental variant, a continuous decrease of phycobiliproteins took place, except in the third cycle, in which the content slightly increased compared to the first and second cycles. The most pronounced decrease was in the fourth cycle, 39% compared to control. In the third experimental variant, the decrease of phycobiliproteins in the first two cycles was accompanied by an increase in the next two cycles. The change in the content of phycocyanin followed the same trend as the case of phycobiliproteins. The most pronounced decrease was noticed in the fourth cycle of the second experimental variant, by 40% compared to control. The allophycocyanin content in the first and second experimental variants was on the level of control or higher (by 14–22%). In the third experimental variant, an abnormal decrease of allophycocyanin by 50% in the second cycle was observed, while in the other cycles, it was higher than control (by 7–22%).

The performed experiments demonstrated that *A. platensis* could be considered a candidate for wastewater treatment on an industrial scale. The biomass’s ability to grow for several cycles in metal contaminated media allows the reduction of operational costs for the process. Further studies are required to develop equipment that could provide, in automatic mode, the cultivation of *A. platensis*, wastewater supply, and removal of the treated water.

## 4. Conclusions

The possibility of applying cyanobacteria *A. platensis* to treat nickel containing mono and polymetallic effluents was tested. Biomass was able to grow, except for the first experimental variant of the Ni/Cu/Zn/Mo system, for four cycles in a metal-loaded medium. The rate of metal uptake by biomass depended on the chemical composition and metal concentrations of the effluents. The highest amount of nickel was accumulated in biomass grown in the Ni/Cr/Fe and Ni/Cu/Sr/Zn systems. Nickel accumulation inhibited to a different extent the uptake of copper, strontium, zinc, and molybdenum by spirulina biomass. The negative effects of the analyzed systems on biomass productivity and protein content were observed mainly in the first cycle of biomass growth. Recovery of biomass and protein content in the next cycles indicated biomass adaptation to new growth conditions. Phycobiliproteins were mainly affected by the action of metal ions. The significant decrease of phycocyanin compared to allophycocyanin confirmed metal affinity concerning the former. *A. platensis* can be considered a potential bioremediator for wastewater treatment. However, its bioaccumulation capacity is strongly dependent on the chemical composition of wastewater.

## Figures and Tables

**Table 1:** Composition of synthetic effluents.

Experimental Variant	Systems	Metal Concentration, mg/L						
		Ni	Cu	Sr	Zn	Cr	Fe	Mo
1	Ni	2.5 ± 0.07	-	-	-	-	-	-
	Ni/Cu/Sr/Zn	2.5 ± 0.07	0.5 ± 0.01	1.0 ± 0.01	0.5 ± 0.01	-	-	-
	Ni/Cr/Fe	2.5 ± 0.07	-	-	-	1.0 ± 0.05	1.0 ± 0.05	-
	Ni/Cu/Zn/Mo	2.5 ± 0.07	1.0 ± 0.01	-	0.5 ± 0.01	-	-	0.5 ± 0.01
2	Ni	5.0 ± 0.1	-	-	-	-	-	-
	Ni/Cu/Sr/Zn	5.0 ± 0.1	1.0 ± 0.05	2.5 ± 0.03	1.0 ± 0.005	-	-	-
	Ni/Cr/Fe	5.0 ± 0.1	-	-	-	2.5 ± 0.01	2.5 ± 0.011	-
	Ni/Cu/Zn/Mo	5.0 ± 0.1	2.5 ± 0.03	-	1.0 ± 0.02	-	-	0.5 ± 0.01
3	Ni	10.0 ± 0.1	-	-	-			-
	Ni/Cu/Sr/Zn	10.0 ± 0.3	1.0 ± 0.02	5.0 ± 0.03	2.0 ± 0.03	-	-	-
	Ni/Cr/Fe	10.0 ± 0.3	-	-	-	5.0 ± 0.1	5.0 ± 0.1	-
	Ni/Cu/Zn/Mo	10.0 ± 0.3	5.0 ± 0.05	-	2.0	-	-	0.5 ± 0.01

## Data Availability

Not applicable.
